# Are smart older adult care devices “cost-reducing and efficiency-enhancing” or “demand-activating”?—Empirical evidence from Chinese older adult households

**DOI:** 10.3389/fpubh.2026.1798799

**Published:** 2026-04-16

**Authors:** Lin Guo, Linlin Zhang, Ming Zhang, Shanna Li, Ying Liu

**Affiliations:** 1 School of Management, Shandong Second Medical University, Weifang, Shandong, China; 2 School of Basic Medical Sciences, Shandong Second Medical University, Weifang, Shandong, China; 3 School of Humanities and Management, Zhejiang Chinese Medical University, Hangzhou, Zhejiang, China

**Keywords:** aging, four-way decomposition, health and care expenditure, smart health devices, technological expansion

## Abstract

**Background:**

Against the backdrop of global aging and the growing burden of chronic diseases, Smart Health Devices (SHD) are regarded as important tools for health management. However, there is a theoretical controversy between “technological substitution” and “technological expansion” regarding their economic consequences: Does SHD reduce medical expenditures through prevention, or push up costs by detecting hidden diseases? Currently, there is a lack of systematic empirical evidence based on large-scale microdata.

**Methods:**

Using data from the 2020 China Longitudinal Aging Social Survey (CLASS) (*N* = 7,426), this study constructed dual indicators of household health and care expenditure (HCE) and its share in total consumption. Ordinary Least Squares (OLS), quantile regression, and the Heckman two-step method were employed to examine the robust association between SHD use and medical expenditure, and the Discacciati four-way decomposition method was used to reveal its intrinsic mechanism.

**Results:**

The study found that the use of SHD was significantly associated with higher absolute household health and care expenditure (by approximately 101%) and a larger share in total consumption (by 1.0 percentage points), supporting the “medical demand activation” hypothesis. Mechanism analysis revealed a unique “dual path”: on the one hand, device use exhibited a positive correlation with total expenditure, likely linked to a higher detection rate of chronic diseases and increased attention to pain (discovery effect); on the other hand, it showed a marginal negative association with cost levels, potentially reflecting improved disease management efficiency (management effect). Heterogeneity analysis showed that “rigid care devices” represented by smart wheelchairs were strongly associated with a heavier dual burden, while “preventive management devices” represented by smart wristbands were significantly correlated with a lower share of medical expenditure. In addition, highly educated and urban groups exhibited stronger expenditure sensitivity, indicating potential digital health inequality.

**Conclusion:**

At the current stage, smart devices mainly act as a “health radar,” corresponding to the explicit manifestation of potential health risks into medical consumption. Policymakers should implement a classified subsidy strategy: include rigid care devices in long-term care insurance payment to prevent catastrophic expenditure, and promote preventive devices through incentive mechanisms to achieve an economic transformation from “passive medical care” to “active health.”

## 1 Introduction

In the global context of increasingly severe population aging, the extension of life expectancy and declining fertility rates have jointly reshaped the population structure. It is predicted that by 2030, approximately one-sixth of the world’s population will be aged 60 and above; by 2070, the number of people aged 65 and above is expected to increase to 2.2 billion, exceeding the number of children aged 18 and below for the first time (1, 2). However, although the life expectancy of the older adults has generally increased, their health span has not grown synchronously, meaning that the additional years of life are often accompanied by the challenges of health decline (1, 2). The World Health Organization (WHO) points out that the number of new cases of chronic diseases among the global older adults is expected to jump from 12.7 million in 2008 to 21.4 million in 2030 (3). With the extension of life expectancy, the risk of non-communicable diseases that require long-term monitoring and management, such as diabetes, kidney failure, arthritis, and Alzheimer’s disease, has increased significantly (4).

These chronic diseases have become the core factors driving the growth of medical expenses for the older adults worldwide (5). In China, medical expenditures of the older adults already account for more than 25% of the national total medical expenditures, among which the proportion of chronic disease expenditures for people aged 60 and above is as high as 48.84% (6). The heavy disease burden not only threatens the sustainability of medical insurance funds but also exposes families to huge economic risks. Guo, He, and Zheng (7) verified that data from both the World Health Organization and the World Bank show that the global incidence of catastrophic health expenditure is on the rise (8), Chen, Zhao, Zhou, Ou, and Yao (9) leading to a large number of families falling into poverty every year due to unbearable burdens (10). In this context, how to seek cost-effective health management solutions has become an urgent global problem.

Technological progress and the demand for chronic disease management have jointly promoted the rapid development of the Smart Health Devices (SHD) market (1, 2). Existing studies have shown that the traditional medical model is difficult to meet the growing health needs. Tan, Sumner, Wang, and Wenjun Yip (11) reviewed remote monitoring, data analysis, and personalized intervention, smart devices provide a feasible solution for efficient and continuous health management, and also reduce the burden on formal and informal caregivers (12). However, despite the significant health value of smart devices, their popularity among the older adults is not optimistic. A study by Guo et al. (7) shows that the usage rate of smart health devices among Chinese older adults is only 6.03%, and the lack of digital literacy and the additional economic pressure brought by the devices are important reasons hindering their popularity (13).

More importantly, when evaluating the impact of smart devices, existing literature mainly focuses on health outcomes such as disease prevention and behavioral intervention, while the discussion on their economic outcomes is insufficient and the conclusions are controversial. The traditional “technological substitution hypothesis” holds that smart devices can reduce medical expenditures by preventing diseases and reducing medical visits; however, the classic health economics theory—“technological expansion hypothesis” points out that the introduction of new technologies often pushes up total medical costs by discovering undiagnosed diseases (“diagnostic expansion”) and providing more expensive treatment methods (14). For a developing country like China, is the popularization of smart devices a “cost-saving tool” or an “inducement to spend money”? Is its intrinsic mechanism “replacing traditional medical care” or “activating potential demand”? At present, there is a lack of systematic demonstration based on large-scale representative data.

To fill the above research gaps, this paper systematically evaluates the relationship between the use of smart health devices and the health and care expenditures (including absolute expenditure and expenditure share) of older adults households based on data from the 2020 China Longitudinal Aging Social Survey (CLASS). The marginal contributions of this study are as follows: First, it fills the empirical gap. This paper provides national evidence from the world’s largest older adults group, correcting the previous research conclusions based only on small samples or specific diseases. Second, it reveals the dual mechanism behind the “high-cost paradox.” Different from the simple expectation of “technological cost reduction,” we find that smart devices push up absolute expenditure through the “discovery effect” (increasing disease detection rate), but also marginally optimize the expenditure structure through the “management effect” (improving chronic disease management efficiency) and “prevention effect” (such as smart wristbands). This mechanism analysis based on the four-way decomposition method (15) provides a new theoretical perspective for understanding the complex relationship between technology and costs. Third, it provides precise policy references. By distinguishing the heterogeneous impacts of “rigid care devices” (such as wheelchairs) and “preventive management devices” (such as wristbands), this study provides a scientific basis for the reform of medical insurance payment systems and the classified promotion of digital health products.

The structure of this paper is arranged as follows: The second part elaborates on the research design in detail, including data sources, variable definitions, and research methods; the third part presents the results of empirical analysis, including benchmark regression, mechanism testing, and subgroup analysis; the fourth part discusses the core findings; finally, the conclusion summarizes the research findings and puts forward policy recommendations, and points out the limitations of this study.

## 2 Research design

### 2.1 Data source

The data in this paper is derived from the “China Longitudinal Aging Social Survey (CLASS),” which is jointly designed and implemented by the Center for Population and Development Studies and the Institute of Gerontology of Renmin University of China. The survey adopts a stratified multi-stage probabilistic sampling method, selects samples nationwide, and systematically collects detailed information on the health status, family structure, socioeconomic background, and care resources of the Chinese older adult group, which has a high national representativeness. This paper selects the latest released 2020 fourth round survey data of CLASS as the basis for analysis.

### 2.2 Cleaning process

The original sample size of the CLASS 2020 survey is 11,398 people. To ensure the logical consistency of the data and the accuracy of the analysis, this study strictly implemented the following sample screening steps: Step 1: Exclude samples with invalid codes in the household health and care expenditure data. Since the original data codes “do not know,” “refuse to answer” and other situations as 999,996, 999,998, or 999,999, these values are seriously deviated from the normal range of household expenditure and have no economic significance. Therefore, we excluded 3,926 samples with monthly average household health and care expenditure exceeding 990,000 yuan, retaining 7,472 samples. Step 2: Exclude logically abnormal samples. According to economic common sense, a certain sub-expenditure of a household should not exceed the total household expenditure. Therefore, we excluded 46 samples with “health and care expenditure share” greater than 1. After the above screening, the final valid analysis sample included in the empirical model is 7,426.

### 2.3 Variable definitions

#### 2.3.1 Dependent variables

This study constructs two core indicators to comprehensively evaluate the economic burden of older adult households:

*Household health and care expenditure (HCE)*: Considering that smart devices often have both medical monitoring and rehabilitation care functions, this study aggregates “monthly average household medical expenses” and “monthly average household housework and rehabilitation care expenses,” collectively referred to as household health and care expenditure. In view of the right-skewed distribution of expenditure data, we performed logarithmic transformation ln(HCE + 1) in the regression analysis.

*Health and care expenditure share (HCE Share)*: Defined as the proportion of monthly average household health and care expenditure to monthly average total household consumption expenditure. This indicator reflects the crowding-out effect of health-related expenditure on other household consumption (such as food and education), and is an important dimension to measure household economic vulnerability.

#### 2.3.2 Core independent variable

**Figure 1:**
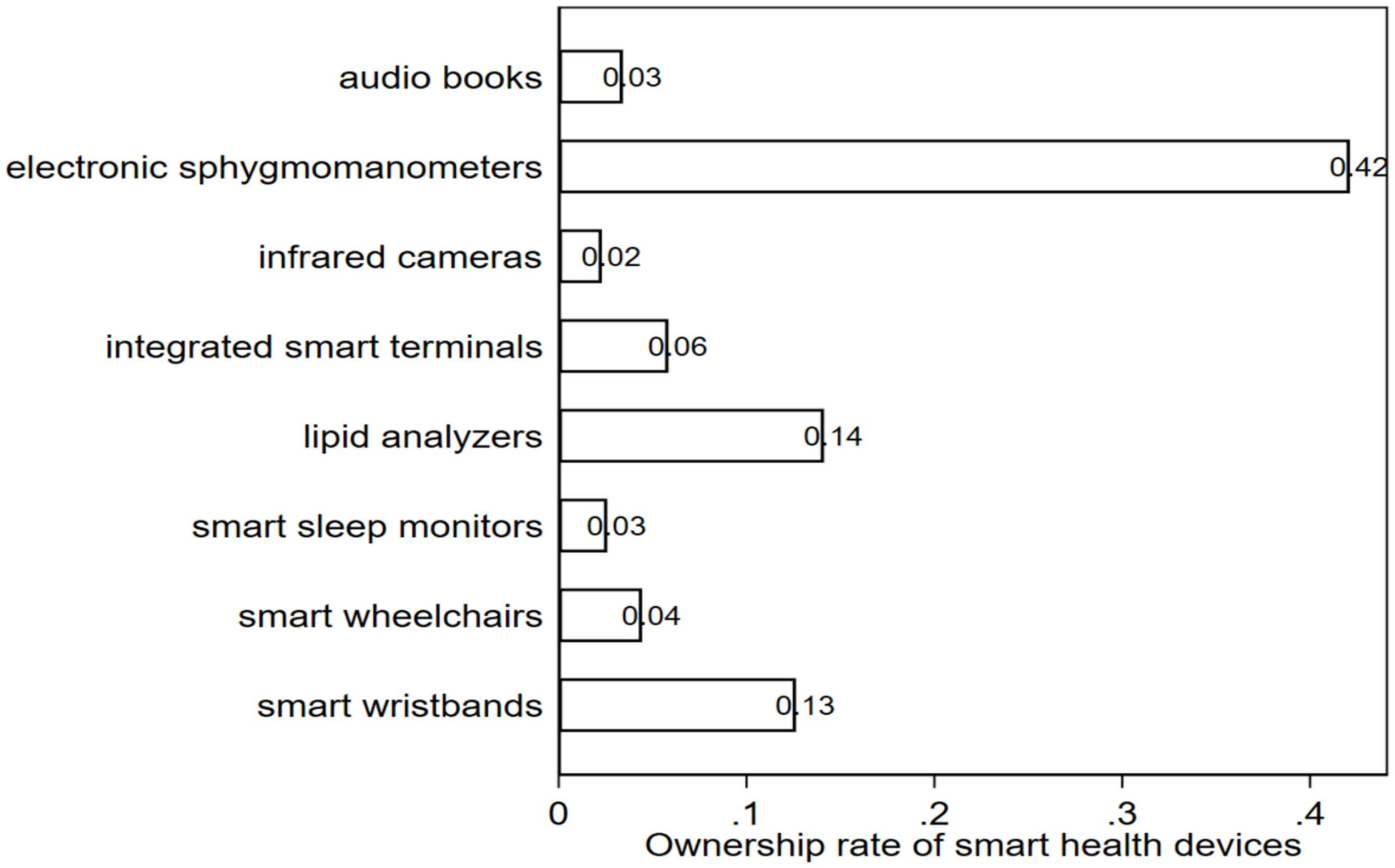
Ownership rate of smart health devices.

*Smart health devices (SHD)*: The core independent variable of this paper. We selected eight representative types of devices in the questionnaire, covering monitoring, rehabilitation, and life support functions, specifically including: smart wheelchairs, electronic sphygmomanometers, lipid analyzers, smart wristbands, infrared cameras, integrated smart terminals, smart sleep monitors, and audio books. We set the variable as a binary variable: if the respondent owns any one or more of the above devices, SHD is assigned a value of 1, otherwise 0. Figure 1 below shows the distribution of the ownership rate of various devices.

#### 2.3.3 Covariates

Referring to Andersen and Newman's (16) behavioral model of medical and health service utilization, this study includes three groups of confounding factors that may affect medical expenditure:

*Predisposing factors*: Including age, gender, marital status, and educational level.

*Enabling factors*: Including type of residence (urban and rural) and household registration status.

*Need and health behaviors*: Including smoking status, etc. The specific definitions and descriptive statistics of each variable are shown in Table 1. To test the problem of multicollinearity, we drew a heat map of variable correlation coefficients (see Appendix). The results show that the correlation coefficients between variables are all at a low level, indicating that there is no serious multicollinearity risk in the model.

**Table 1:** Univariate analysis.

Variable	Total	Non-users	Users	*χ*^2^/*t*
	(*N* = 7,426)	(*N* = 3,958)	(*N* = 3,468)	
Residence (%)				
Rural	3,108 (41.85)	2,073 (52.38)	1,035 (29.84)	385.56***
Urban	4,318 (58.15)	1,885 (47.63)	2,433 (70.16)	
Gender (%)				
Female	3,683 (49.60)	1,875 (47.37)	1,808 (52.13)	16.76***
Male	3,743 (50.40)	2,083 (52.63)	1,660 (47.87)	
Education (%)				
Primary or below	4,705 (63.36)	2,895 (73.14)	1,810 (52.19)	365.10***
Secondary	2,525 (34.00)	1,013 (25.59)	1,512 (43.60)	
Tertiary or above	196 (2.64)	50 (1.26)	146 (4.21)	
Marital status (%)				
Unmarried	1,822 (24.54)	1,069 (27.01)	753 (21.71)	28.00***
Married	5,604 (75.46)	2,889 (72.99)	2,715 (78.29)	
Smoking (%)				
Non-smoker	5,382 (72.48)	2,887 (72.94)	2,495 (71.94)	0.92
Smoker	2,044 (27.52)	1,071 (27.06)	973 (28.06)	
Hukou (%)				
Urban	3,840 (51.71)	1,476 (37.29)	2,364 (68.17)	705.62***
Agricultural	3,586 (48.29)	2,482 (62.71)	1,104 (31.83)	
HCE (ln)	7,426 (5.51)	5.09 (1.60)	6.00 (1.22)	<0.001***
HCE Share	7,426 (0.19)	0.18 (0.14)	0.19 (0.13)	0.083
Age	7,426 (71.70)	71.89 (6.78)	71.49 (6.99)	<0.001***

SHD = smart health devices. For categorical variables, values represent frequencies (*n*) with column percentages (%) in parentheses. For continuous variables, values are means with standard deviations in parentheses. ***p < 0.01.

**Figure 2:**
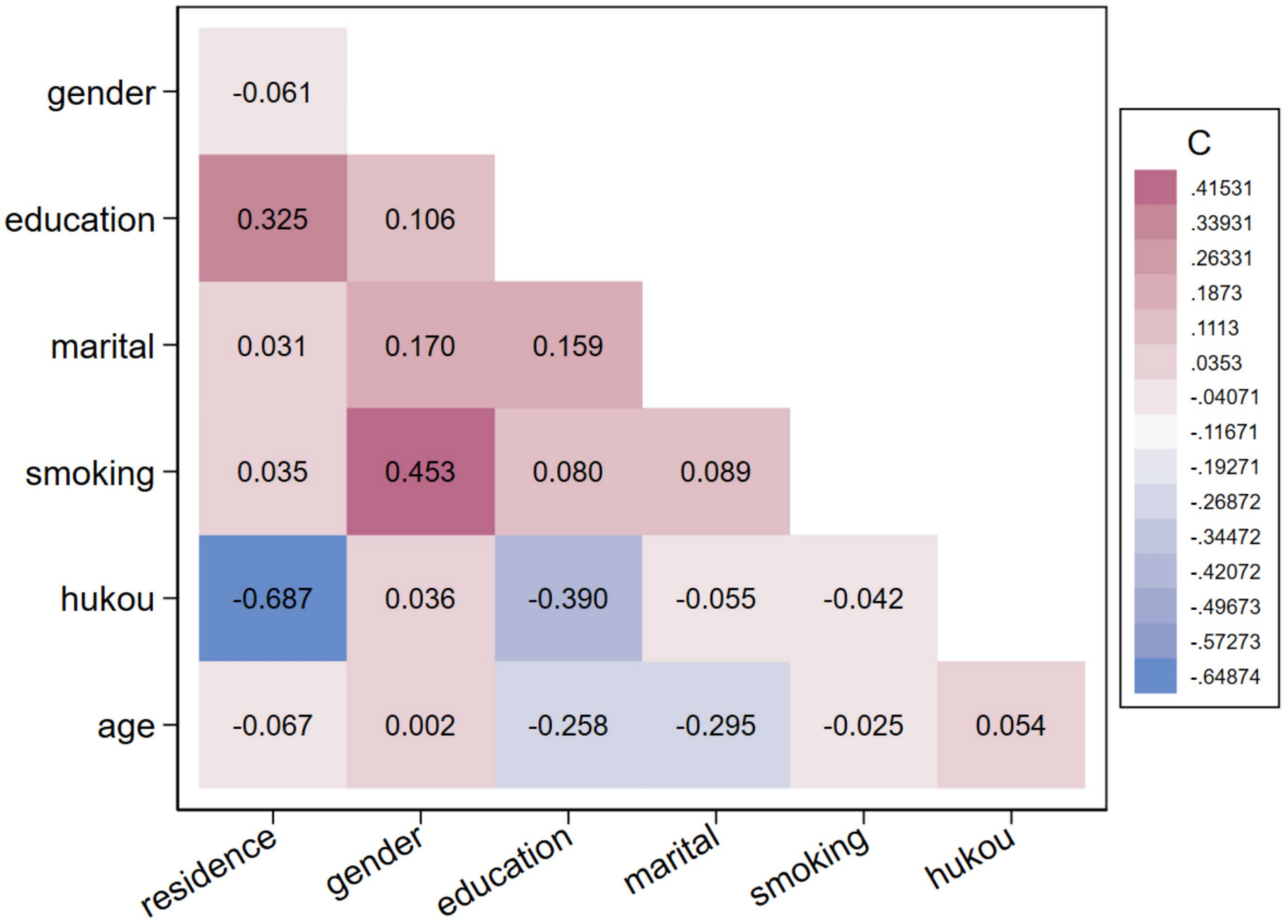
Pearson correlation coefficient heatmap of covariates. The values in the figure represent the Pearson correlation coefficients between variables. The depth of color represents the strength of correlation, red represents positive correlation, and blue represents negative correlation. The specific definitions and codes of each variable are as follows: residence (type of residence: 1 = urban, 0 = rural); gender (gender: 1 = male, 0 = female); education (educational level: 1 = primary school and below, 2 = secondary school, 3 = college and above); marital (marital status: 1 = married, 0 = unmarried); smoking (smoking status: 1 = smoking, 0 = non-smoking); hukou (household registration status: 1 = non-agricultural household registration, 0 = agricultural household registration); age (continuous variable).

To avoid the interference of multicollinearity on the regression results, we calculated the Variance Inflation Factor (VIF) and correlation coefficient matrix between covariates. The results in Figure 2 below show that the VIF values of all variables are far below 10, and most of the pairwise correlation coefficients are at a low level, indicating that there is no serious multicollinearity problem in the model.

### 2.4 Research methods

This study follows a standardized econometric paradigm and conducts empirical analysis in four steps. First, univariate analysis is used to initially test the association between core variables and characteristic variables, where *t*-test is used for continuous variables and Chi-square test is used for categorical variables. Second, a multiple linear regression (Ordinary Least Squares, OLS) model is constructed as the benchmark regression to evaluate the average effect of the use of smart health devices on household health and care expenditure (logarithmic form) and health and care expenditure share, respectively. Third, to verify the robustness of the results and handle potential endogeneity issues, this study implements multi-dimensional sensitivity analysis: (1) Propensity Score Matching (PSM) method is used to mitigate selection bias of observable variables by constructing a counterfactual framework; (2) LASSO regression model is used to test the sensitivity of the results to the selection of control variables; (3) Heckman two-step method is used to correct potential sample self-selection bias. In addition, considering that mean regression may mask differences at both ends of the distribution, this study further uses quantile regression to examine the heterogeneous impact of smart devices on households with different expenditure levels. Fourth, in the mechanism analysis, this study goes beyond the traditional mediation effect model and adopts the four-way decomposition method proposed by Discacciati et al. (15). This method decomposes the total effect (TE) into four parts: Pure Indirect Effect (PIE) (mediation path), Mediated Interaction (INTMED) (the device changes the degree of influence of the mediator variable on expenditure), Reference Interaction (INTREF), and Controlled Direct Effect (CDE). This method can simultaneously identify the mediation path and the interaction between the smart device and the mediator variable, thereby providing a deeper mechanism explanation. Finally, this study conducts heterogeneity analysis for eight specific types of devices and subsamples with different demographic characteristics. All empirical results in this paper are calculated using Stata 17.0 software.

## 3 Results

### 3.1 Univariate analysis

Table 2 reports the descriptive statistical results of the full sample and subsamples. Among the 7,426 surveyed older adults, 3,468 (46.7%) used at least one smart health device (SHD = 1), and the remaining 3,958 (53.3%) did not (SHD = 0). Univariate analysis (*t*-test and Chi-square test) shows that there are significant differences in multiple characteristics between the device-using group and the non-using group: In terms of core outcome variables, older adults households using smart devices have significantly higher logarithmic health and care expenditure (mean: 6.00 vs. 5.09, *p* < 0.001); although the health and care expenditure share (HCE Share) of the using group is slightly higher than that of the non-using group (0.19 vs. 0.18), the difference does not reach the 0.05 significance level at the univariate level (*p* = 0.083). In terms of demographic and sociological characteristics, smart device users show significant socioeconomic advantages. Compared with non-users, users have a younger average age (71.49 years vs. 71.89 years, *p* < 0.001), and the proportion of those with secondary school education or above is significantly higher (47.81% vs. 26.85%). It is worth noting that the distribution of household registration and residence is highly consistent: the proportion of users with non-agricultural (urban) household registration is as high as 68.17%, much higher than 37.29% of the non-using group (*p* < 0.001); similarly, more users live in urban areas (70.16% vs. 29.84%). In addition, a higher proportion of users are married (78.29% vs. 72.99%), and the proportion of females is slightly higher (52.13% vs. 47.37%).

**Table 2:** Association between smart health devices and health and care expenditure.

Variables	ln (Expenditure)		Share	
	Coefficient	95%CI	Coefficient	95%CI
SHD (1 = Yes)	0.70***	0.636 to 0.770	0.01***	0.008 to 0.020
Residence (1 = Urban)	0.28***	0.187 to 0.364	−0.00	−0.009 to 0.007
Gender (1 = Male)	−0.01	−0.081 to 0.064	−0.00	−0.010 to 0.004
Education	0.19***	0.125 to 0.259	−0.00	−0.009 to 0.004
Marital Status (1 = Married)	0.16***	0.083 to 0.239	0.01	−0.001 to 0.013
Smoking (1 = Yes)	−0.22***	−0.304 to −0.145	−0.00	−0.012 to 0.003
Hukou (1 = Urban)	0.34***	0.253 to 0.435	−0.02***	−0.031 to -0.014
Age	0.03***	0.029 to 0.039	0.00***	0.003 to 0.004
Observations	7,426	7,426	7,426	7,426

SHD = smart health devices. CI = confidence interval. The reference groups for categorical variables are: Residence (Rural), Gender (Female), Education (Primary or below), Marital (Unmarried), Smoking (Non-smoker), Hukou (Non-agricultural/Urban). ***p < 0.001.

### 3.2 Association between smart health devices and household health care expenditure

Table 3 reports the benchmark regression results regarding the association between the use of smart health devices and household health care expenditure and its share in total household consumption (HCE Share). All models control for confounding factors such as demographic, socioeconomic, and health behaviors.

**Table 3:** Sensitivity analysis results.

	ln (Expenditure)		Share	
Methods	Coefficient	95% CI	Coefficient	95% CI
PSM (ATT)	0.84***	0.717 to 0.959	0.02***	0.017 to 0.023
Lasso	0.35	–	0.01	–
Heckman	0.70***	0.634 to 0.768	0.01***	0.008 to 0.020
Single-living older adults	0.78*	0.494 to 1.058	0.03**	0.007 to 0.055
Empty-nest couples	0.55*	0.463 to 0.640	0.01*	0.007 to 0.023

SHD = smart health devices. PSM estimates represent the Average Treatment Effect on the Treated (ATT) using 1:2 nearest neighbor matching. Lasso coefficients are selected based on the optimal lambda. Heckman estimates account for selection bias. *p < 0.1, **p < 0.05, ***p < 0.001.

In terms of absolute health and care expenditure [ln (Expenditure)], the regression results show that the use of smart health devices is significantly and positively associated with monthly average household health and care expenditure (Coefficient = 0.70, *p* < 0.001). From an economic perspective, holding other factors constant, older adults households using smart devices have a monthly average health and care expenditure that is approximately 101.3% higher than that of non-users (e^0^·^70^ – 1 ≈ 1.013). This large magnitude of association indicates that the use of smart devices is often accompanied by more intensive use of medical services or care input, rather than a simple substitution effect.

In terms of health and care expenditure share (Share), the use of smart health devices is also significantly associated with a higher household medical economic burden (Coefficient = 0.01, *p* < 0.001). This means that the proportion of health and care expenditure in total consumption for households using smart devices is, on average, 1.0 percentage point higher than for non-users. Although the coefficient seems small, considering that the average share of the sample is only 19% (see Table 2), this difference is not negligible, indicating that the ownership of smart devices is correlated with a certain crowding-out effect on other household consumption.

In terms of covariates, socioeconomic characteristics are significantly associated with health and care expenditure. Older adults living in urban areas (Coefficient = 0.28), with higher educational levels (Coefficient = 0.19), and married (Coefficient = 0.16) have significantly higher absolute health and care expenditure. Household registration status (Hukou) shows significant structural differences: taking non-agricultural household registration as the reference group, older adults with agricultural household registration have significantly lower absolute health and care expenditure (Coefficient = −0.34, *p* < 0.001), which is about 71% of that of non-agricultural household registration; however, their health and care expenditure share is significantly higher (Coefficient = 0.02, *p* < 0.001), about 2.0 percentage points higher. In addition, smokers have significantly lower absolute health and care expenditure than non-smokers (Coefficient = −0.22, *p* < 0.001).

### 3.3 Robustness tests

To verify the reliability of the above benchmark regression results, this study conducted sensitivity analysis in four dimensions: (1) Propensity Score Matching (PSM) method was used to mitigate selection bias of observable variables; (2) Lasso regression method was used to test the dependence of the model on the selection of control variables; (3) Heckman two-step method was used to correct potential sample self-selection bias; (4) Subsample analysis was conducted to restrict the sample to pure older adult households, eliminating potential confounding from multi-generational family structures.

First, Propensity Score Matching (PSM). Considering that the use of smart devices is not randomly assigned, there may be systematic differences in baseline characteristics between the using group and the non-using group. We used 1:2 nearest neighbor matching to balance the covariates. The model was re-estimated in the matched sample (see Table 3). The results show that.

the coefficient for the association between smart health devices and household health care expenditure (ln HCE) is 0.84 (*p* < 0.001), and the coefficient for expenditure share is 0.02 (*p* < 0.001). This indicates that after eliminating the differences in observable characteristics between groups, the positive association between smart devices and medical burden remains robust, and the magnitude of this association is slightly larger.

Second, Lasso regression. To exclude the interference of multicollinearity and specific combinations of control variables on the results, we introduced the Lasso (Least Absolute Shrinkage and Selection Operator) algorithm for variable selection and regression. This method automatically eliminates redundant variables by applying L1 regularization penalty. The results show (Table 3) that in the model after Lasso screening, the positive association between smart devices and health care expenditure (Coeff = 0.35) and expenditure share (Coeff = 0.01) remains significant, proving that the core conclusion does not depend on specific model settings.

Third, Heckman two-step method. In view of the fact that some older adults may take the initiative to choose to use devices based on their own health status (such as unobserved frailty), leading to endogeneity issues, we used the Heckman selection model for correction. The first step constructs a Probit selection equation to predict the probability of device use, and the second step introduces the calculated Inverse Mills Ratio (IMR) into the main regression equation. The results show (Table 3) that after controlling for potential selection bias, the positive correlation between smart devices and health care expenditure (Coeff = 0.70, *p* < 0.001) and expenditure share (Coeff = 0.01, *p* < 0.001) is highly consistent with the benchmark regression, further confirming the robustness of the results.

Fourth, Sample Restriction for Expenditure Alignment. A potential limitation of using household-level expenditure data is the confounding effect of non-older adults family members in multi-generational households, whose medical costs might be inherently included in the total household health and care expenditure. To accurately isolate the economic association of smart devices specifically on the older adults, we restricted our analysis to two strictly defined subsamples: “single-living older adults households” (*N* = 708) and “empty-nest couple households” (*N* = 3,867). Re-estimating the baseline models revealed that the positive associations remained robust. For single-living older adult, SHD use was robustly associated with higher absolute expenditure (Coeff = 0.78, *p* < 0.001) and a higher expenditure share (Coeff = 0.03, *p* = 0.012). Similar highly significant results were observed for empty-nest couples. This stringent robustness check confirms that our core findings are fundamentally driven by the older adults themselves, rather than the medical consumption of younger household members.

### 3.4 Quantile regression analysis

**Figure 3:**
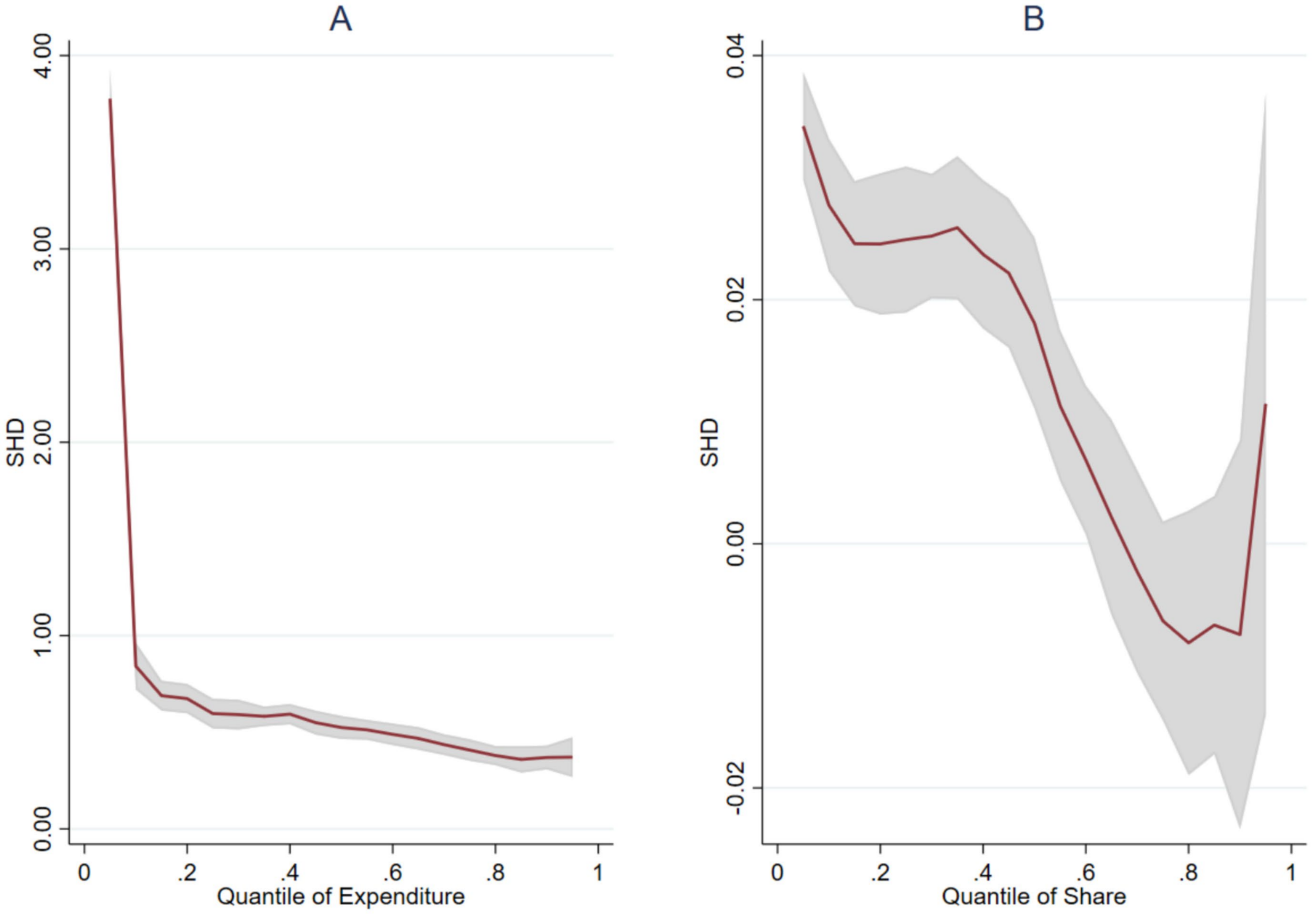
Quantile regression estimates of SHD on HCE and HCE share. The solid red line represents the quantile regression estimates of the SHD coefficient ranging from the 5th to the 95th percentile. The gray shaded area indicates the 95% confidence interval. **(A)** Outcome is logarithm of health and care expenditure (ln HCE). **(B)** Outcome is the share of HCE in total household consumption. Control variables are included as in the baseline model.

Since the benchmark OLS regression can only reflect the “average association” of smart devices with medical expenditure, it may mask the distribution differences between households with different expenditure levels. Therefore, this study uses the quantile regression model to examine the varying correlations between smart health devices and health care expenditure (ln HCE) and expenditure share (HCE Share) at different quantiles. The results are shown in Figure 3.

Figure 3A (health and care expenditure) shows that the regression coefficient of SHD presents a significant “L-shaped” downward trend. At the low quantile of the health and care expenditure distribution (*q* = 0.1), the coefficient is as high as about 4.0, then decreases rapidly, and stabilizes at around 0.5 at the middle and high quantiles (*q* = 0.5–0.9). This indicates that the association between smart device ownership and medical expenditure is strongest among low-expenditure households—for households at the lower end of the medical consumption distribution, device ownership corresponds to a substantial “from zero to one” difference in expenditure (activation effect). For households at the higher end of the expenditure distribution, the expenditure difference associated with smart devices is relatively limited.

Figure 3B (expenditure share) also shows obvious heterogeneity. At the low quantile (i.e., households with light medical burden), smart health devices ownership is significantly associated with a higher expenditure share (coefficient about 0.04); as the quantile rises, the coefficient gradually decreases, and even turns negative near *q* = 0.75 (although the confidence interval becomes wider). This means that smart device ownership is primarily correlated with a larger medical expenditure share among.

households that otherwise have a light medical burden; for households already experiencing a high medical burden, smart devices may instead be associated with a marginally lower relative expenditure, potentially reflecting the substitution of expensive traditional care or improved management efficiency.

### 3.5 Mixed effect analysis of moderating mechanisms

Traditional mediation analysis usually assumes that there is no interaction between the core variable and the mediator variable, which may lead to misjudgment of the effect. To overcome this limitation and deeply explore the complex pathways linking smart health devices ownership to household health care expenditure, this study adopts the four-way decomposition method proposed by Discacciati et al. (15). This method decomposes the overall association (TE) into four components: Pure Indirect Effect (PIE) (mediation path), Mediated Interaction (INTMED) (capturing how device ownership modifies the correlation between the mediator and expenditure), Reference Interaction (INTREF), and Controlled Direct Effect (CDE).

Table 4 shows the decomposition results based on five mechanisms: length of hospital stay, use of medical and nursing services, pain status, number of chronic diseases, and depression score. The analysis reveals that the relationship between smart health devices and health care expenditure presents a unique “discovery-management” dual logic:

**Table 4:** Four-way decomposition results.

Variables	Mechanism	Effect decomposition	Coefficient	95%CI	Effect share
ln (Expenditure)	Mechanism 1: Length of Hospital Stay	Total effect	0.82***	0.713 to 0.924	
		Pure indirect effect	0.74***	0.635 to 0.842	90.2%
		Reference interaction	−0.02***	−0.037 to −0.008	−2.4%
		Mediated interaction	0.05***	0.023 to 0.083	6.1%
		Controlled direct effect	0.05***	0.029 to 0.070	6.1%
SHARE	Mechanism 1: Length of Hospital Stay	Total effect	0.03***	0.024 to 0.046	
		Pure indirect effect	0.03***	0.017 to 0.039	100.0%
		Reference interaction	−0.00**	−0.003 to −0.001	
		Mediated interaction	0.00**	0.001 to 0.008	
		Controlled direct effect	0.00***	0.002 to 0.006	
ln (Expenditure)	Mechanism 2: Use of Medical and Nursing Services	Total effect	0.70***	0.637 to 0.771	
		Pure indirect effect	0.66***	0.595 to 0.726	94.3%
		Reference interaction	0.00	−0.002 to 0.007	
		Mediated interaction	−0.01	−0.016 to 0.004	
		Controlled direct effect	0.05***	0.030 to 0.062	7.1%
SHARE	Mechanism 2: Use of Medical and Nursing Services	Total effect	0.01***	0.008 to 0.021	
		Pure indirect effect	0.01***	0.005 to 0.017	100%
		Reference interaction	0.00*	0.000 to 0.001	
		Mediated interaction	−0.00***	−0.002 to 0.000	
		Controlled direct effect	0.00***	0.003 to 0.005	
ln (Expenditure)	Mechanism 3: Pain Status	Total effect	0.71***	0.640 to 0.776	
		Pure indirect effect	0.65***	0.581 to 0.713	91.5%
		Reference interaction	0.01***	0.002 to 0.015	1.4%
		Mediated interaction	−0.02***	−0.032 to −0.007	−2.8%
		Controlled direct effect	0.07***	0.051 to 0.093	9.9%
SHARE	Mechanism 3: Pain Status	Total effect	0.01***	0.007 to 0.020	
		Pure indirect effect	0.01**	0.003 to 0.016	100%
		Reference interaction	0.00***	0.001 to 0.002	
		Mediated interaction	−0.00***	−0.004 to −0.001	
		Controlled direct effect	0.00***	0.004 to 0.008	
ln (Expenditure)	Mechanism 4: Number of Chronic Diseases	Total effect	0.70***	0.634 to 0.768	
		Pure indirect effect	0.61***	0.542 to 0.672	87.1%
		Reference interaction	0.01***	0.006 to 0.020	1.4%
		Mediated interaction	−0.03***	−0.052 to −0.017	−4.3%
		Controlled direct effect	0.12***	0.092 to 0.139	17.1%
SHARE	Mechanism 4: Number of Chronic Diseases	Total effect	0.01***	0.008 to 0.020	
		Pure indirect effect	0.01**	0.002 to 0.015	100%
		Reference interaction	0.00***	0.000 to 0.002	
		Mediated interaction	−0.00***	−0.005 to −0.002	
		Controlled direct effect	0.01***	0.006 to 0.009	100%
ln (Expenditure)	Mechanism 5: Depression Score	Total effect	0.70***	0.636 to 0.77	
		Pure indirect effect	0.72***	0.653 to 0.787	102.9%
		Reference interaction	0.00	−0.002 to 0.005	
		Mediated interaction	0.00	−0.011 to 0.005	
		Controlled direct effect	−0.02***	−0.024 to −0.008	−2.9%
SHARE	Mechanism 5: Depression Score	Total effect	0.01***	0.007 to 0.020	
		Pure indirect effect	0.01***	0.009 to 0.022	100%
		Reference interaction	0.00***	0.001 to 0.002	
		Mediated interaction	−0.00***	−0.004 to −0.001	
		Controlled direct effect	-0.00	−0.001 to 0.000	

SHD = smart health devices. The decomposition allows for interactions between SHD and the mediator. PIE represents the effect mediated solely through the mediator. INTMED captures the interaction effect caused by the mediator level changing due to exposure. INTREF captures the interaction effect when the mediator is fixed at the reference level. *p < 0.1, **p < 0.05, ***p < 0.001.

First, “Discovery and Access Correlation”: Length of hospital stay and use of medical and nursing services serve as significant positive mediators between smart health devices and health care expenditure (PIE accounts for 6.1 and 7.1% respectively). This indicates that the use of smart devices ownership is associated with greater older adults attention to their own health status, and the monitoring of health data corresponds to a higher frequency of utilizing, thereby correlating with higher expenditure. The pure indirect effect (PIE) of the number of chronic diseases accounts for as high as 17.1% (0.12, *p* < 0.001). This means that smart devices are strongly linked to a higher detection rate of chronic diseases (early detection), which corresponds to a significantly higher level of treatment costs in the short term.

Second, “Management and Efficiency Correlation”: Although the detection of chronic diseases and pain is associated with higher expenditure, the decomposition results show that the mediated interaction effects (INTMED) of the number of chronic diseases and pain status are negative (accounting for −4.3% and −2.8% respectively). This reveals a profound mechanism: for older adults people who are already ill or in pain, the use of smart devices ownership is correlated with better disease management efficiency through continuous monitoring and intervention, thereby being marginally associated with a lower relative level of excessive growth of costs. The reference interaction effect (INTREF) of length of hospital stay is negative (−2.4%), indicating that for the same length of hospital stay, patients using smart devices exhibit slightly lower costs, potentially due to more accurate monitoring substituting for redundant medical examinations.

Third, “Mental Health Dividend”: The depression score acts as a significant negative mediator (PIE accounts for −2.9%). This indicates that smart devices (such as companion-type or sleep monitoring devices) are associated with better mental health status among the older adults, which in turn correlates with fewer depression-induced somatization symptoms and lower corresponding medical expenditure.

### 3.6 Subgroup analysis

**Figure 4:**
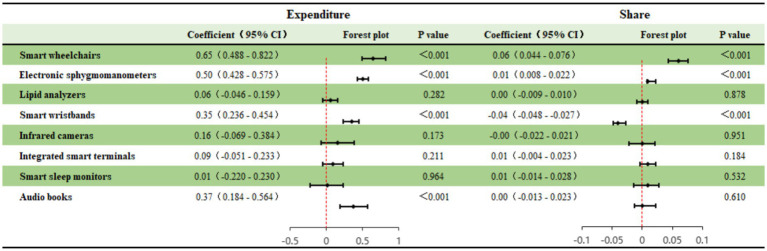
Heterogeneous impacts of different smart health devices.

To decompose the specific correlates of smart devices regarding medical burden, this study conducted regression analysis for eight types of devices with different functions (see Figure 4). The results show that different types of devices have significant structural differences in their association with health care expenditure (Expenditure) and expenditure share (Share), showing the characteristics of coexisting “care-associated higher costs” and “management-correlated relative burden reduction.”

First, rigid care and monitoring devices are significantly associated with a heavier dual burden. Smart wheelchairs and electronic sphygmomanometers show significant positive associations with both absolute expenditure and expenditure share. Among them, smart wheelchairs exhibit the strongest correlation (Expenditure Coeff = 0.65; Share Coeff = 0.06), followed by electronic sphygmomanometers. This indicates that such devices are usually highly bound to the rigid care needs of disability or chronic diseases (such as hypertension). Their ownership is not only accompanied by high equipment and maintenance costs but also often corresponds to more frequent use of medical services.

Second, health management devices show potential for “relative burden mitigation.” Although smart wristbands are positively correlated with absolute expenditure (Coeff = 0.35, which may be linked to equipment purchase costs), they show a significant negative correlation in the expenditure share (Share) model (Coeff = −0.04, *p* < 0.001). This is a very valuable finding, meaning that for older adults who use wearable devices for active health management, although their absolute consumption is slightly higher, the relative proportion of their medical expenditure in the family budget is, on average, 4.0 percentage points lower. This may be attributed to the preventive health care function of smart wristbands, which correlates with a lower risk of serious diseases, thereby potentially reflecting a lower likelihood of catastrophic medical expenditure.

Third, the association of other devices is limited. Audio books are only correlated with higher absolute expenditure (Coeff = 0.37) and have no significant association with the share, which may reflect more the consumption of auxiliary equipment for the visually impaired group. Lipid analyzers, infrared cameras, integrated smart terminals, and sleep monitors did not show robust statistical significance in either model, which may be related to their low popularity among the older adults or unclear functional positioning.

**Figure 5:**
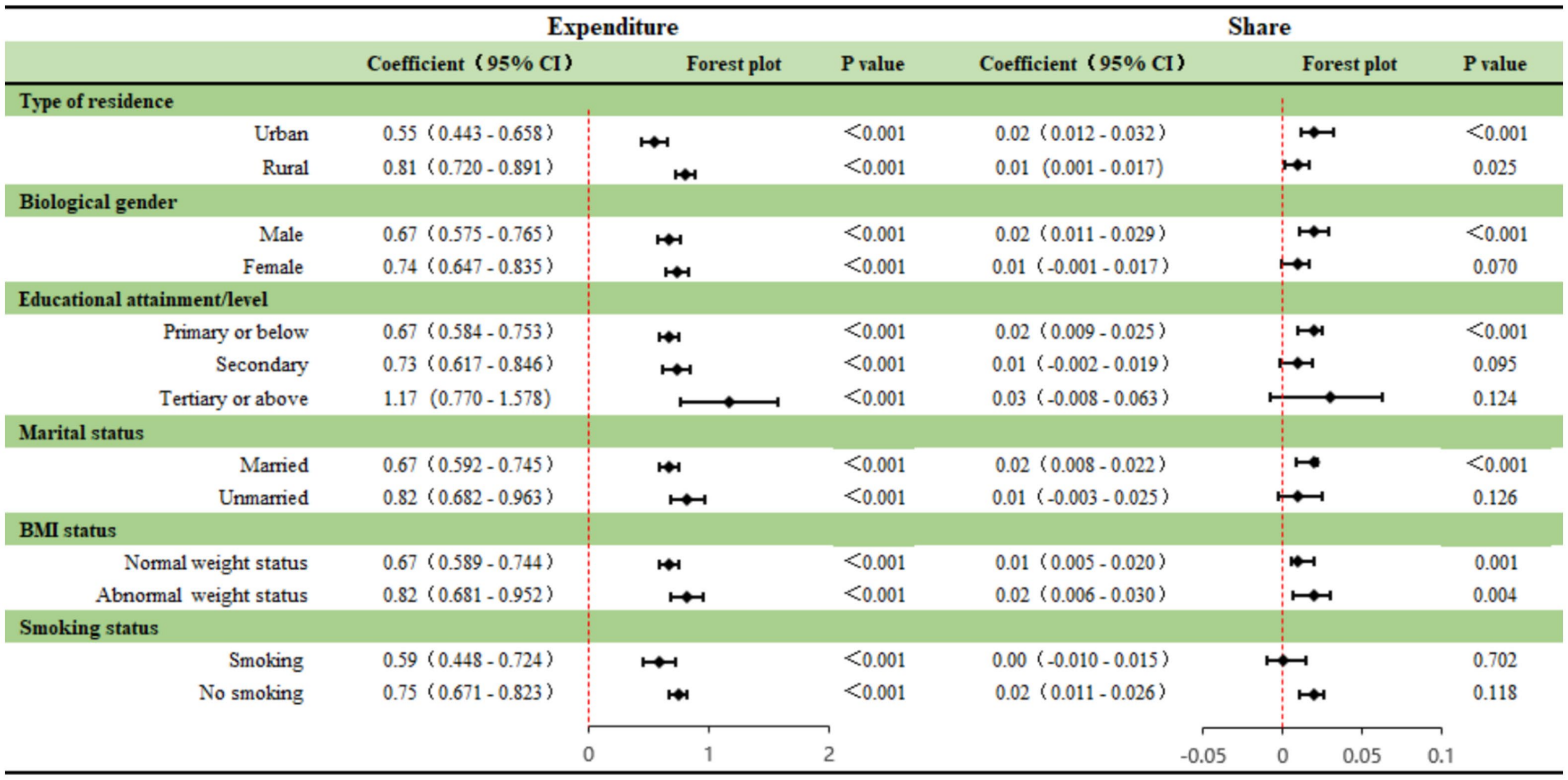
Heterogeneous impacts of smart health devices by demographic characteristics.

To explore the heterogeneity in the association between smart health devices ownership and medical burden across different subpopulations, this study conducted a subgroup analysis based on six key demographic characteristics (see Figure 5). The results reveal significant structural differences in these correlations among different social groups:

First, the correlations related to socioeconomic status (SES) present a pattern akin to a “Matthew Effect.” Educational level shows a strong gradient: as educational level increases, the positive association between smart health devices and absolute health care expenditure becomes markedly stronger (the coefficient is 0.67 for primary school and below, compared to 1.17 for college and above). This suggests that highly educated groups with smart devices also exhibit a stronger propensity for higher concurrent medical investments, potentially reflecting a stronger “knowledge-attitude-behavior” alignment. Urban–rural differences reveal distinct resource constraint contexts: the absolute expenditure coefficient for rural residents (0.81) is significantly larger than that for urban residents (0.55), suggesting that in rural areas with relatively scarce medical resources, device ownership is more strongly correlated with the manifestation of underlying medical needs (an “enlightenment” correlation). However, the expenditure share coefficient for urban residents is higher (0.02 vs. 0.01), potentially reflecting a more pronounced relative financial burden on the family budget under the pressure of urban living costs.

Second, family structure correlates point toward a potential “care substitution” context. The grouping results by marital status show that the health and care expenditure coefficient for unmarried (including widowed/divorced) older adults (0.82) is significantly higher than that for married older adults (0.67). This indicates that in the absence of spousal care resources, smart device ownership is associated with even higher medical and service expenditures, potentially reflecting their role as a “substitute care resource.”

Third, heterogeneity exists across different health awareness and demand indicators. The expenditure coefficient for the group with abnormal weight (BMI Abnormal) (0.82) is notably higher than that for the group with normal weight (0.67), aligning with the “medical demand-driven hypothesis”—that is, higher health risks correspond to a stronger correlation between device ownership and disease management expenditures. Interestingly, the expenditure coefficient for non-smokers (0.75) is significantly higher than that for smokers (0.59). This supports the “Health Consciousness Hypothesis”: non-smokers, who typically exhibit stronger health literacy, show a stronger positive association between device ownership and preventive or diagnostic medical investments.

## 4 Discussion

Within the scope of this study sample, the use of smart health devices was significantly associated with a higher level of medical expenditure, which is contrary to the initial research hypothesis.

Based on nationally representative data in China, this study reveals the complex association between Smart Health Devices and household health care expenditure of the older adults. The study found that at the current stage, the use of smart devices is significantly correlated with higher household health and care expenditure (absolute values are approximately twice as high, and the share is 1.0 percentage point larger). This “high-cost paradox” challenges the traditional assumption that technology will inevitably bring about “cost reduction and efficiency improvement.”

First, smart devices align more closely with the role of a “medical demand activator” rather than a simple cost substitute. Our benchmark regression results show that the use of smart health devices is closely related to substantially higher expenditure, which is consistent with the conclusion of De Guzman et al. (17) that remote monitoring devices may be associated with higher medical costs in the short term. This higher expenditure level is likely linked not only to equipment purchase costs (18) but also to the device’s role as a “health radar,” which makes hidden health problems (such as hypertension fluctuations and arrhythmia) explicit through continuous monitoring, thereby correlating with “from zero to one” medical behaviors and inspection expenditures. This “discovery correlation” is positive to a certain extent because it corresponds to the translation of potential health risks into explicit medical interventions, even if it manifests as a heavier family economic burden concurrently.

Second, the four-way decomposition reveals the dual mechanism of “discovery correlates with higher expenditure” and “management correlates with optimized burden reduction.” Through detailed mechanism decomposition, this study corrects the one-sided view of the single role of devices in previous studies. On the one hand, we found that the pure indirect effects (PIE) through the number of chronic diseases and pain status are significantly positive. This means that device ownership corresponds to the older adults seeking more medical services alongside a higher detection rate of diseases and attention to health, thereby correlating with higher expenditure. This explains why the total association is positive. On the other hand, the mediated interaction term (INTMED) is significantly negative. This indicates that for older adults who have already been diagnosed or are in pain, smart devices are associated with an effective “management dividend”—through real-time monitoring and precise intervention (such as improved compliance as described by (3)), device ownership is marginally associated with a mitigation of the higher expenditures typically linked to deterioration of the condition. In addition, the negative mediating path of the depression score confirms the potential of devices in psychological comfort and mental health management (19). This “mental health dividend” is correlated with fewer somatization-related medical needs caused by emotional problems. The negative reference interaction (INTREF) of length of hospital stay further suggests that device-assisted monitoring may substitute for unnecessary redundant inspections during hospitalization and reflect improved efficiency of in-hospital services.

Third, the heterogeneity of device types reveals the economic logic differences between “rigid care” and “preventive investment.” Not all devices are “money-burning.” Our subgroup analysis found a key dividing line: “rigid care devices” represented by smart wheelchairs and electronic sphygmomanometers are strongly bound to high expenditure. This is usually because users of such devices are mostly disabled or chronically ill (such as hypertension), and the devices themselves are a sign of high-frequency medical consumption. It is crucial to define the scope of these specific findings: since devices like smart wheelchairs have a relatively low population-level prevalence (around 4%), their strong association with higher medical costs reflects the severe economic burden concentrated specifically within highly vulnerable subgroups with rigid long-term care needs, rather than a universal phenomenon applicable to the general healthy older adults.

Finally, differences in demographic characteristics reflect a “Matthew Effect” and “resource compensation” in the era of digital health. The stronger expenditure correlation among high-educated groups (coefficient 1.17) indicates that groups with higher socioeconomic status exhibit a stronger “knowledge-attitude-behavior” alignment and can more effectively make health investments based on device feedback, which may highlight potential health inequalities. Interestingly, the absolute expenditure coefficient of rural older adults (0.81) is higher than that of urban older adults (0.55), indicating that in rural areas with relatively scarce medical resources, smart device ownership may correspond more strongly to an “enlightenment” role, correlating with the manifestation of underlying medical needs. The high expenditure coefficient of unmarried groups suggests a “technical compensation” context for devices in the absence of family care (20), which correlates with higher purchase and service expenditures. In addition, non-smokers and people with abnormal weight have higher expenditure sensitivity (21, 22), indicating that health awareness and objective risks jointly correlate with rational, data-driven medical consumption.

## 5 Conclusion

Based on nationally representative data in China, this study systematically evaluates the complex association between Smart Health Devices and the health care expenditure of older adults households and their budget share. The main conclusions are as follows:

First, on the whole, smart devices align with the profile of a “medical demand activator,” but they also show structural “burden reduction” potential. Benchmark regressions show that the use of smart health device is significantly associated with higher absolute household health and care expenditure and its share, which verifies the applicability of the “technological expansion theory” in the field of digital health. However, the heterogeneity analysis by device type reveals a key dividing line: “rigid care devices” represented by smart wheelchairs and electronic sphygmomanometers are significantly correlated with a heavier dual burden, reflecting the rigid costs of disability and chronic disease management; while “preventive management devices” represented by smart wristbands, although associated with higher absolute expenditure, are significantly correlated with a lower share of health and care expenditure in total household consumption. This indicates that through active health behavior interventions (such as exercise and sleep management), preventive technology shows potential to correspond with an optimized family consumption structure and achieve relative burden reduction.

Second, demographic characteristics are closely linked to the distribution of “digital dividends.” The study found that groups with higher socioeconomic status (urban, highly educated, married) exhibit a stronger correlation between device monitoring and actual health investments, showing higher expenditure levels. This suggests that digital health technology currently corresponds more strongly to the “health upgrading” of advantaged groups rather than “providing timely help” to vulnerable groups. Without intervention, this may highlight or widen potential health inequalities.

Third, the underlying pathways suggest a dual logic of “discovery correlates with costs, and management correlates with improved efficiency.” The four-way decomposition results show that device ownership corresponds to the greater use of medical services alongside a higher detection rate of chronic diseases and attention to pain (positive pure indirect effect), thereby correlating with higher total expenditure. However, at the same time, devices are associated with a significant efficiency improvement role in the continuous disease management stage (negative mediated interaction effect), marginally corresponding to a mitigation of excessive cost growth. In addition, the positive association between devices and the amelioration of depressive mood also confirms their implicit economic value in the field of mental health.

Based on the above findings, this study puts forward the following policy recommendations: (1) Implement a “classified and precise subsidy” strategy. In view of the heterogeneity of device economic profiles, medical insurance policies should be differentiated: for “rigid care devices” (such as smart wheelchairs) that are bound to a heavier burden, it is recommended to include them in the long-term care insurance payment scope to alleviate the risk of catastrophic expenditure for families with disabilities. For “preventive management devices” (such as smart wristbands) that show potential for reducing relative burden, exploring incentives through health points or commercial insurance discounts can promote them as low-cost tools for “preventing diseases before they occur.” (2) Bridge the “digital health divide.” For digitally vulnerable groups such as the rural older adults, the less-educated older adults, and the older adults living alone, simple device donations are insufficient. Communities should rely on service centers to carry out age-appropriate digital skills training, improving their ability to use device data for self-health management, thereby preventing technical dividends from flowing exclusively to advantaged groups. (3) Promote data integration for a “monitoring-diagnosis-treatment” closed loop. To maximize the “management efficiency” function of devices, it is recommended to break down data barriers between home monitoring and clinical medical care. Encouraging primary health care institutions to access the data interfaces of mainstream smart devices will allow medical staff to conduct precise interventions based on continuous monitoring data. This can reduce unnecessary medical visits caused by “excessive anxiety” and truly realize the economic transformation from “passive medical care” to “active health.”

This study still has the following limitations: First, limited by its cross-sectional data nature, this study cannot fully establish a strict causal relationship because it is inherently unable to determine the definitive temporal sequence between device adoption and changes in medical expenditure. Although we employed Propensity Score Matching (PSM) and the Heckman two-step method to mitigate observable and unobservable selection biases to the greatest extent possible, these statistical strategies cannot completely substitute for the rigorous causal identification power of a longitudinal design. Therefore, the current findings should be prudently interpreted as robust associations rather than definitive causal effects. Future research needs to rely on longitudinal tracking data to verify the long-term dynamic effects of device use. Second, the device use data relies on self-reporting, which may introduce measurement errors. Third, while the absolute sample size for specific devices like smart wheelchairs is statistically sufficient for regression analysis, their low relative ownership rates (e.g., 4%) dictate that the economic associations observed for these specific devices represent the dynamics of highly specific, high-need demographic segments. These conclusions should be applied prudently and avoid over-generalization. Finally, with the emergence of new technologies such as generative AI, the types of devices covered by the existing questionnaire may lag behind market developments. Future research can further combine objective device data and experimental designs to explore the economic consequences of emerging technologies under different health behavior patterns.

## Data Availability

Publicly available datasets were analyzed in this study. This data can be found here: the data underlying this study are from the China Longitudinal Aging Social Survey (CLASS). The official data application portal is available at http://jkzgyjy.ruc.edu.cn/sjzy/CLASSsjsq/ade1ba3977354553a798c8f10221c2ad.htm. A portion of the CLASS data (2016–2018) is also archived in the OpenICPSR repository under Project ID 191983 (DOI: 10.3886/E191983V1).

## Author’s note

SL and YL should be considered co-corresponding authors.
